# Evaluation of the Role of Tanshinone I in an In Vitro System of Charcot-Marie-Tooth Disease Type 2N

**DOI:** 10.3390/ijms252011184

**Published:** 2024-10-17

**Authors:** Jingjing Zhang, Xinru Meng, Qianni Qin, Yali Liang, Guangpu Yang, Shen Li, Xiaorong Li, Ji-Chang Zhou, Litao Sun

**Affiliations:** 1 School of Public Health (Shenzhen), Shenzhen Campus of Sun Yat-sen University, Shenzhen 518107, China; zhangjj75@mail2.sysu.edu.cn (J.Z.); mengxr6@mail2.sysu.edu.cn (X.M.); qinqn@mail2.sysu.edu.cn (Q.Q.); liangyali2023@163.com (Y.L.); yangguangpu183@163.com (G.Y.); lish376@mail2.sysu.edu.cn (S.L.); lixr36@mail2.sysu.edu.cn (X.L.); 2 Shenzhen Key Laboratory of Pathogenic Microbes and Biosafety, Shenzhen 518107, China

**Keywords:** alanyl-tRNA synthetase, Charcot Marie Tooth disease, Tanshinone I

## Abstract

Charcot-Marie-Tooth disease type 2N (CMT2N) is an inherited nerve disorder caused by mutations in the alanyl-tRNA synthetase (AlaRS) gene, resulting in muscle weakness and sensory issues. Currently, there is no cure for CMT2N. Here, we found that all five AlaRS mutations in the aminoacylation domain can interact with neuropilin-1 (Nrp1), which is consistent with our previous findings. Interestingly, three of these mutations did not affect alanine activation activity. We then performed a high-throughput screen of 2000 small molecules targeting the prevalent R329H mutant. Using thermal stability assays (TSA), biolayer interferometry (BLI), ATP consumption, and proteolysis assays, we identified Tanshinone I as a compound that binds to and modifies the conformation of the R329H mutant and other CMT-related AlaRS mutants interacting with Nrp1. Molecular docking and dynamic simulation studies further clarified Tanshinone I’s binding mode, indicating its potential against various AlaRS mutants. Furthermore, co-immunoprecipitation (Co-IP) and pull-down assays showed that Tanshinone I significantly reduces the binding of AlaRS mutants to Nrp1. Collectively, these findings suggest that Tanshinone I, by altering the conformation of mutant proteins, disrupts the pathological interaction between AlaRS CMT mutants and Nrp1, potentially restoring normal Nrp1 function. This makes Tanshinone I a promising therapeutic candidate for CMT2N.

## 1. Introduction

CMT (Charcot-Marie-Tooth disease) is the most common inherited peripheral neuropathy, with a prevalence of approximately 17.69/100,000 [1,2,3]. The main clinical features of CMT include slowly progressive, symmetrical distal muscle weakness, length-dependent sensory impairment, and musculoskeletal deformities, all of which contribute to significant disability and a reduced quality of life (QoL) [4]. CMT disease exhibits significant genetic heterogeneity, and to date, more than 100 genes have been identified associated with CMT [5,6]. CMT disease can be divided into three main types: autosomal dominant inheritance (demyelinating [CMT1] and axonal [CMT2]), X-linked (CMTX1), and autosomal recessive inheritance [7]. Currently, there are no effective pharmacological treatments for CMT, leaving rehabilitation therapy and surgery for skeletal deformities as the only available options [8,9].

Aminoacyl-tRNA synthetases (aaRSs) are among the most significant protein families associated with CMT, with six members identified so far, including glycyl-, tyrosyl-, tryptophanyl-, alanyl-, histidyl-, and methionyl-tRNA synthetases [10]. Research has shown that RNA interference (RNAi) targeting mutant aaRS mRNA almost completely prevents neuropathies in mice when administered at birth. This suggests that inhibiting mutant aaRS expression, thereby eliminating their toxic gain-of-function, could be a potential treatment strategy for CMT caused by aaRS mutations [11]. In addition to gene therapy, which targets the expression of mutant proteins at the genetic level, small molecule therapies may also be a viable option. These molecules could be designed to either inhibit the stability of mutant proteins or modulate the cellular pathways that regulate their expression. By directly targeting mutant proteins or their regulatory pathways, small molecules offer a practical and potentially less invasive alternative to gene therapy, contributing to the development of more comprehensive and effective treatment strategies.

aaRS have been implicated in disease through conformational changes that result in mutant protein structures and the acquisition of novel abnormal functions. A well-documented example is the competitive binding of aaRS mutants to neuropilin-1 (Nrp1), a receptor for vascular endothelial growth factor (VEGF). This interaction disrupts normal signaling pathways and contributes to disease progression [10,12,13]. By intervening in aaRS conformational changes with small molecule drugs, it may be possible to reduce their abnormal functions, thereby alleviating or treating CMT diseases.

This study focuses on AlaRS and investigates the abnormal binding between AlaRS mutants and Nrp1. Specifically, the R329H mutant, which is the most severe and widely distributed, was selected as the primary target for drug screening. By employing TSA (thermal stability assay), BLI (biolayer interferometry), and proteolysis assays, the small molecule drug Tanshinone I was identified to alter the conformation of R329H and other AlaRS aminoacylation domain mutants that bind to Nrp1. Furthermore, molecular docking and kinetic simulation experiments were conducted to analyze the mechanism of action and potential binding sites of Tanshinone I. These results indicate that Tanshinone I effectively targets and modifies the conformation of CMT-related AlaRS aminoacylation domain mutants, thereby disrupting their abnormal binding with Nrp1. Additionally, at both the cellular and biochemical levels, we validated the disruptive effect of Tanshinone I on the interaction between CMT-related AlaRS mutants and Nrp1. These findings provide a crucial molecular basis for the small molecule drug-based treatment of CMT2N diseases, demonstrating the potential of targeting conformational changes to intervene in mutant protein functions. This lays a solid foundation for developing more effective treatment strategies and may advance the treatment of CMT2N and other similar diseases.

## 2. Results

### 2.1. Identification of AlaRS Mutations Related to CMT and Their Interaction with Nrp1

**Figure 1:**
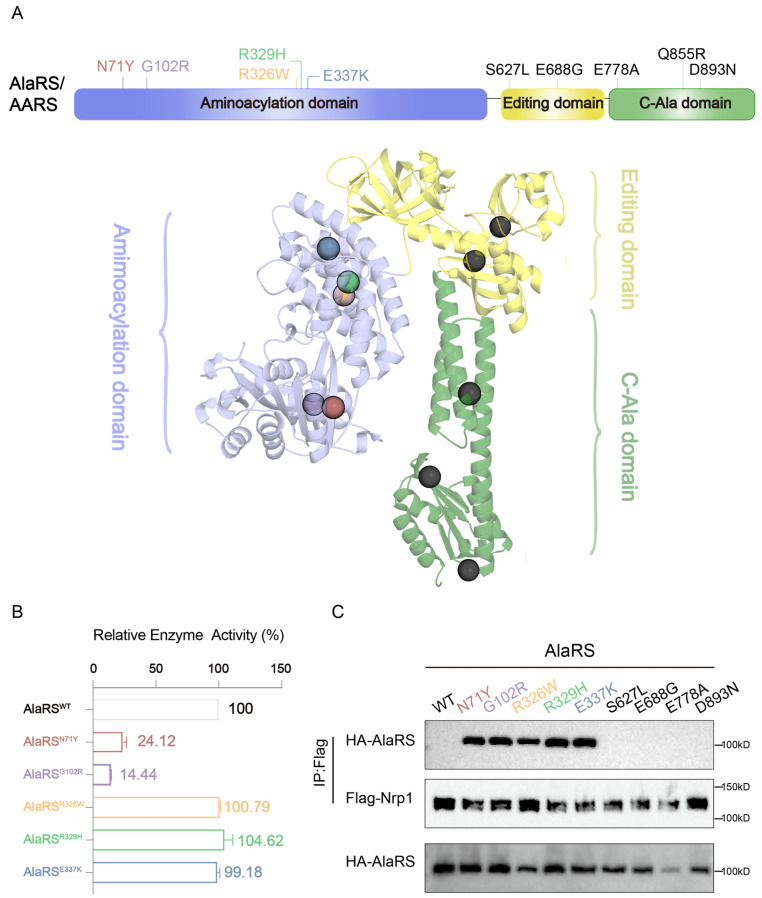
Distribution of CMT-causing mutations on AlaRS and abnormal interactions with Nrp1. (**A**) Distribution of CMT-causing mutations on AlaRS. Ten CMT2N-associated dominant mutations distributed across all three domains of human cytosolic AlaRS. Structure model of AlaRS generated by Alphafold. CMT mutation sites are indicated by colored balls in the aminoacylation domain and black balls in the editing domain and C-Ala domain. (**B**) Enzymatic activity of AlaRS proteins measured by ATP assumption assay with purified enzymes, Alanine, and ATP. The reaction was negatively controlled with Alanine and ATP alone (no enzyme). Data are presented as mean ± SD (n = 2). (**C**) Nrp1-AlaRS interaction was detected by coimmunoprecipitation analysis using anti-HA antibody. AlaRS (WT or CMT2N mutants) was expressed in NSC34 cells with a HA tag detected by an anti-HA antibody. Nrp1 was expressed with a Flag tag detected by an anti-Flag antibody.

Ten AlaRS mutations have been identified in association with the CMT2N subtype [14,15,16,17,18,19,20,21]. Of these, five variants (AlaRS^N71Y^, AlaRS^G102R^, AlaRS^R326W^, AlaRS^R329H^, and AlaRS^E337K^) are located in the aminoacylation domain, two (AlaRS^S627L^ and AlaRS^E688G^) are in the editing domain, and the remaining three (AlaRS^E778A^, AlaRS^Q855R^, and AlaRS^D893N^) are in the C-Ala domain [14,15,16,17,18,19,20,21]. We predicted the full-length structure of AlaRS using Alphafold [22] and specifically marked the positions of the mutations on the model diagram (Figure 1A).

By expressing and purifying five mutations within the aminoacylation domain of AlaRS, we tested their initial enzymatic activity using an ATP-luciferase assay, which measures the activity of AlaRS mutants in amino acid activation. The results showed that the relative enzymatic activity of the N71Y mutant was approximately 24.12%, G102R was 14.44%, R326W was 100.79%, R329H was 104.62% and E337K was 99.18% (Figure 1B). These data indicate that while the enzymatic activities of the N71Y and G102R mutants were reduced, the activities of the other mutants showed no significant difference compared to the wild type.

Our previous studies suggested that AlaRS mutations may cause disease through toxic gain-of-function, leading to abnormal interactions with Nrp1 [12]. To further investigate these interactions, we performed co-immunoprecipitation (Co-IP) experiments, which revealed that the AlaRS mutants N71Y, G102R, R326W, R329H, and E337K exhibited abnormal binding to Nrp1 (Figure 1C). The observed mutations may trigger pathological changes by altering the binding properties of AlaRS, which are key factors in the pathogenesis of CMT2N. Targeting these abnormal interactions with small molecules offers the potential to develop more targeted treatments that could improve clinical outcomes for CMT2N patients.

### 2.2. TSA-Based Small Molecule Screening Targeting AlaRS^R329H^

To identify pharmacological interventions that could mitigate the harmful effects of mutations and alleviate associated pathological symptoms, we selected the R329H mutation as a representative of CMT-related AlaRS mutants in the aminoacylation domain for our drug screening. This choice was based on the prevalence and severity of the R329H mutation, which is the most common disease-causing variant linked to AlaRS [12]. The AlaRS mutations associated with CMT are thought to cause a conformational change that creates a new binding mode, leading to abnormal interactions with Nrp1 and resulting in disease. We therefore performed TSA for small molecule screening to identify leads that bind to the AlaRS mutant.

**Figure 2:**
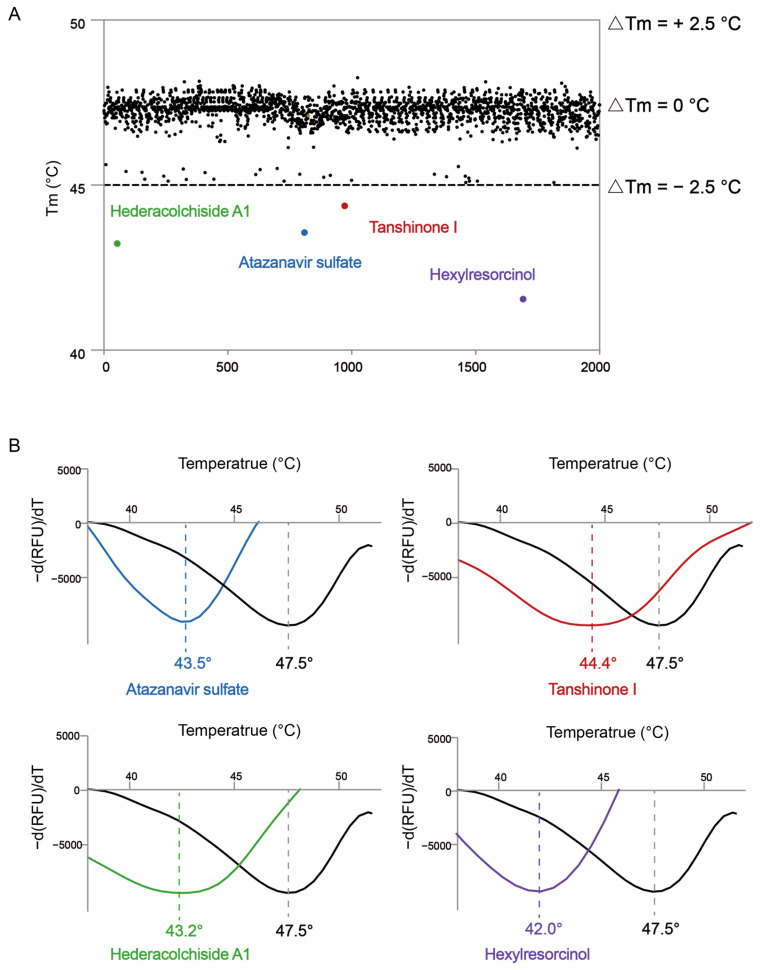
Drug screening based on TSA assay. (**A**) Screening for molecules binding to aminoacylation domain of AlaRS^R329H^ based on the thermal shift assay. A total of four compounds shifted the melting temperature of AlaRS^R329H^ by >2.5 °C and were considered positive hits among the 2000 tested compounds. Black points represent negative hits. (**B**) The interaction analysis of AlaRS^R329H^ and four positive compounds by TSA. The Tm of AlaRS^R329H^ was approximately 47.5 °C and a shift >2.5 °C was observed in the Tm with Atazanavir sulfate, Tanshinone I, Hederacolchiside A1, and Hexylresorcinol.

In this assay, with increasing temperatures, the protein undergoes thermal unfolding, exposing its hydrophobic core. Subsequently, a fluorescent dye binds to these hydrophobic regions, becoming unquenched. Fluorescence is closely monitored, and the midpoint of the protein unfolding transition is designated as the Tm. Ligand binding frequently influences protein thermal stability during denaturation. Thus, the ligand binding affinity of any potential inhibitor can be gauged by evaluating the shift in the Tm. In this experiment, we screened a library of 2000 small molecules (Table S1) and classified as positive hits those that caused a shift in the Tm of the mutant protein of more than 2.5 °C. From the TSA, we identified four small molecules that met this criterion (Figure 2A, Table 1). Specifically, Atazanavir sulfate caused a shift of 4.0 °C, Tanshinone I a shift of 3.1 °C, Hederacolchiside A1 a shift of 4.3 °C, and Hexylresorcinol a shift of 5.5 °C (Figure 2B).

### 2.3. Validating Positive Hits with BLI and ATP Consumption Assays

**Figure 3:**
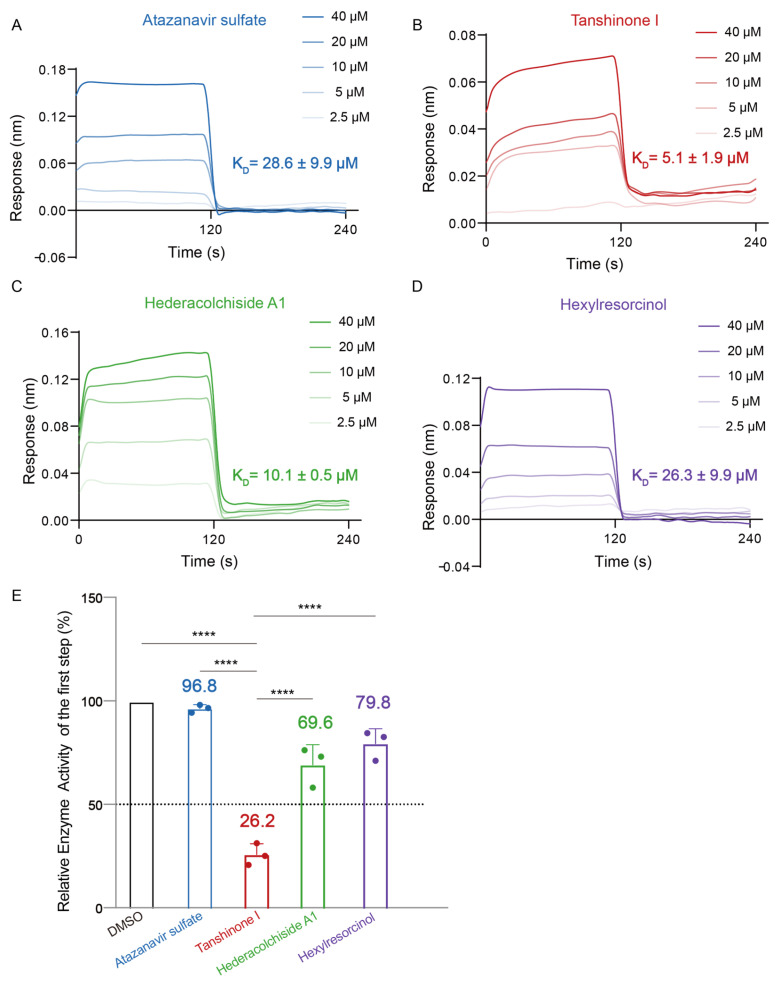
BLI and ATP consumption assays for the four small molecules. Binding sensorgrams for the interaction of Atazanavir sulfate (**A**), Tanshinone I (**B**), Hederacolchiside A1 (**C**), and Hexylresorcinol (**D**) with immobilized AlaRS^R329H^. Each sensorgram contains five curves generated from 40, 20, 10, 5, and 2.5 μM concentrations. The K_D_ values are presented as the mean ± SD and experiments were performed in triplicate. (**E**) The inhibitory effects of the four positive hits were compared with the DMSO control (n = 3). The average relative enzyme activity calculated from three experiments is labelled above the bar graph. An analysis of variance (ANOVA) was conducted to compare the effects of different compounds on relative enzyme activity, followed by post-hoc analyses (Tukey’s test) to account for differences between the treatment groups. ****: *p* < 0.0001.

To quantitatively analyze the binding affinity between the small molecules and the CMT mutant proteins, we performed BLI analysis on the selected candidates. BLI offers direct measurements of drug–protein binding kinetics, including key parameters such as the equilibrium dissociation constant (K_D_), association rate (K_on_), and dissociation rate (K_off_). This analysis provided key insights into the binding affinities of the four small molecules with the mutant protein AlaRS^R329H^. Atazanavir sulfate exhibited a K_D_ value of 28.6 ± 9.9 μM, indicating a low binding affinity (Figure 3A). In contrast, Tanshinone I displayed a K_D_ value of 5.1 ± 1.9 μM, suggesting stronger binding (Figure 3B). Hederacolchiside A1 showed relatively tight binding, with a K_D_ of 10.1 ± 0.5 μM, positioning it as a promising candidate (Figure 3C). Hexylresorcinol showed moderate binding affinity with a K_D_ of 26.3 ± 9.9 μM (Figure 3D).

We further investigated the effects of the four small molecules on the enzymatic activity of the AlaRS^R329H^ protein using an ATP consumption assay. All four compounds affected enzymatic activity to varying degrees. Atazanavir sulfate treatment resulted in 96.8% of the mutant activity, while Tanshinone I caused a significant reduction to 26.2%. Hederacolchiside A1 reduced the activity to 69.6% and Hexylresorcinol to 79.8% (Figure 3E). These results indicate that Tanshinone I exhibits the most pronounced inhibitory effect on the enzymatic activity of the R329H mutant.

### 2.4. Binding and Conformational Effects of Tanshinone I on CMT Mutants

**Figure 4:**
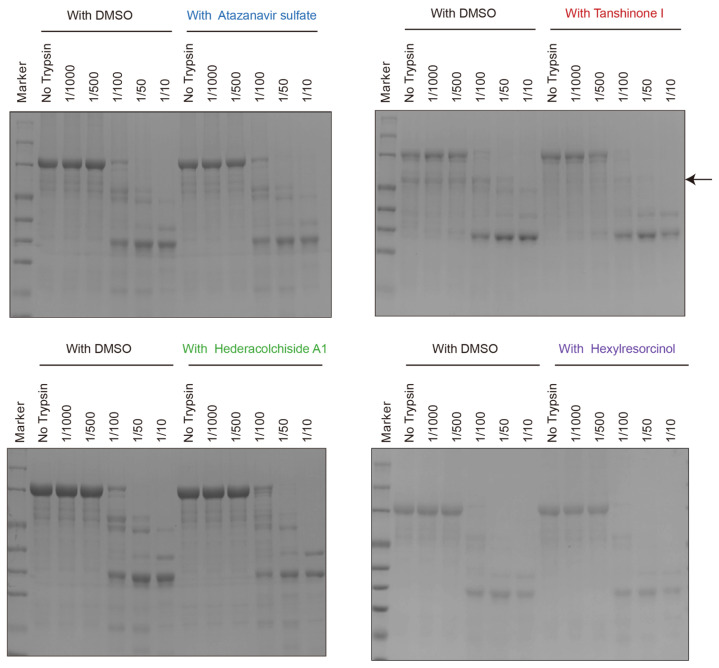
Proteolytic digestion assay of AlaRS^R329H^ with four small molecules. The AlaRS^R329H^ proteins (in the presence and absence of Atazanavir sulfate, Tanshinone I, Hederacolchiside A1, and Hexylresorcinol) were incubated with trypsin at different concentrations for 3 h before the reactions were quenched, and the products were separated by SDS-PAGE. The addition of Tanshinone I resulted in a significant change in the distribution of the bands at the indicated position.

To delve deeper into the impact of selected small molecules on the structural conformation of AlaRS^R329H^ proteins, a series of proteolysis experiments were conducted. The small molecules were incubated with AlaRS^R329H^ proteins at room temperature for three hours, followed by SDS-PAGE analysis to evaluate their effects on the distribution of the digestion fragments. The results showed that Atazanavir sulfate and Hexylresorcinol had minimal effects on the distribution of digestion fragments, while Hederacolchiside A1 caused a slight conformational change in AlaRS^R329H^ (Figure 4). In contrast, Tanshinone I markedly altered the digestion fragment pattern, suggesting that it had a significant effect on AlaRS^R329H^ conformation.

**Figure 5:**
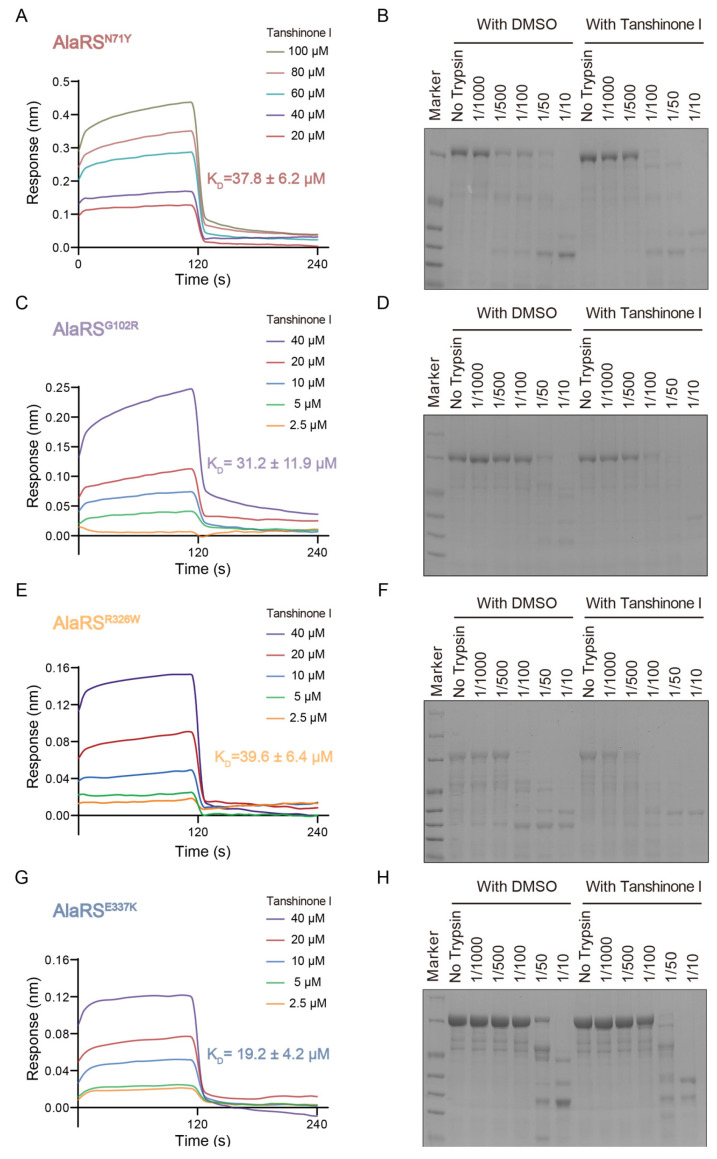
BLI and proteolytic digestion assay of AlaRS mutants with Tanshinone I. Binding sensorgrams for the interaction of Tanshinone I with immobilized AlaRS^R329H^ (**A**), AlaRS^G102R^ (**C**), AlaRS^R326W^ (**E**), and AlaRS^E337K^ (**G**). Each sensorgram contains five curves generated from five different concentrations. The K_D_ values are presented as the mean ± SD and experiments were performed in triplicate. The AlaRS^N71Y^ (**B**), AlaRS^G102R^ (**D**), AlaRS^R326W^ (**F**), and AlaRS^E337K^ (**H**) proteins (in the presence and absence of Tanshinone I) were incubated with trypsin at different concentrations for three hours before the reactions were quenched, and the products were separated by SDS-PAGE.

Furthermore, we examined the interaction of Tanshinone I with additional AlaRS CMT mutants capable of binding to Nrp1 and causing conformational changes by BLI and proteolysis assays on additional related mutants. BLI revealed that Tanshinone I has a moderate binding affinity for the N71Y mutant, with a K_D_ of 37.8 ± 6.2 μM (Figure 5A). Proteolysis assays showed that the N71Y mutant began to exhibit distinct proteolytic fragments at a 1/500 trypsin concentration, with more prominent bands observed at 1/100 after Tanshinone I treatment (Figure 5B), indicating a significant conformational change. For the G102R mutant, BLI yielded a K_D_ of 31.2 ± 11.9 μM (Figure 5C), while proteolysis assays revealed distinct fragments at a 1/50 trypsin concentration, with more prominent bands at 1/100 after Tanshinone I treatment (Figure 5D). The R326W mutant showed a K_D_ of 39.6 ± 6.4 μM (Figure 5E) and distinct proteolytic fragments after Tanshinone I treatment (Figure 5F). The E337K mutant showed a higher binding affinity, with a K_D_ of 19.2 ± 4.2 μM (Figure 5G). Proteolysis assays revealed distinct fragments at a 1/50 trypsin concentration, with altered band patterns after Tanshinone I treatment (Figure 5H). Overall, these findings highlight that Tanshinone I induces significant conformational changes in various AlaRS CMT mutants.

### 2.5. Molecular Docking and Stability Analysis of Tanshinone I with AlaRS Mutants

**Figure 6:**
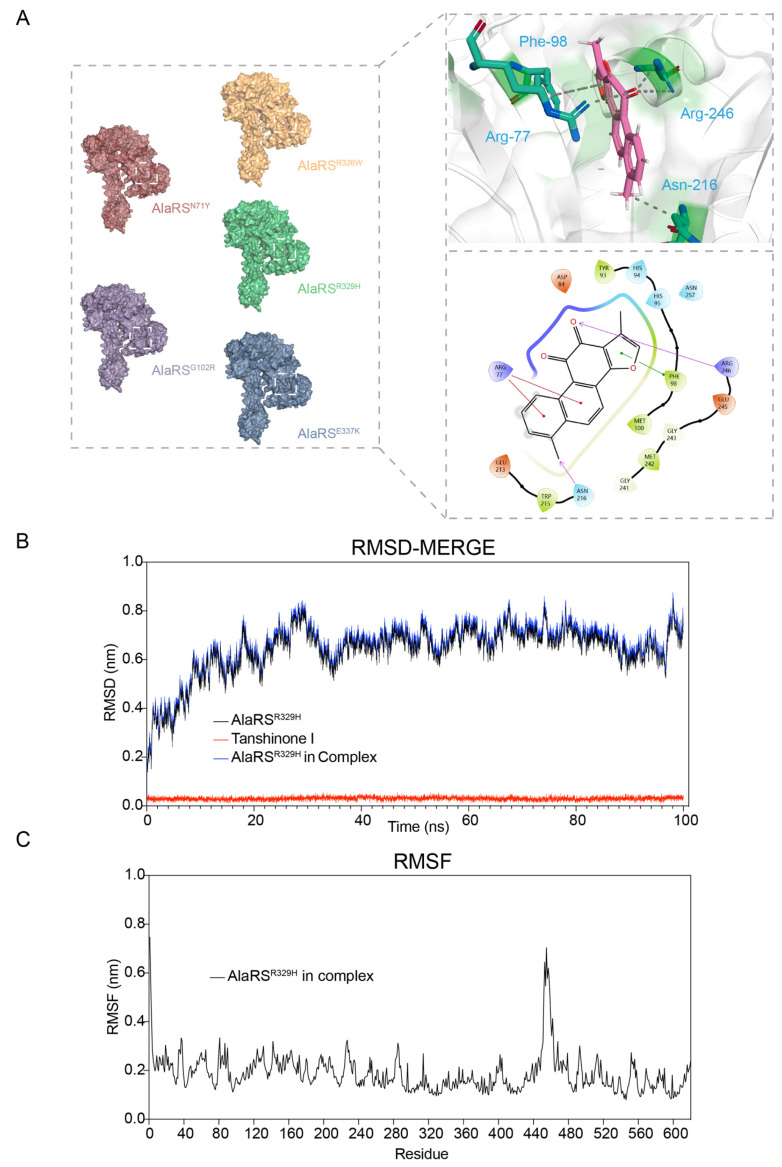
Molecular docking and dynamic simulations of Tanshinone I with AlaRS Mutants. (**A**) The molecular docking model of AlaRS mutants and Tanshinone I was generated by Autodock Vina v.1.2.2. The two-dimensional images were generated and adjusted using Schrödinger’s LigPrep module (Release 2019-2, Schrödinger LLC, New York, NY, USA, 2019) [23]. (**B**) Molecular dynamics (MD) simulations of the AlaRS mutant/Tanshinone I complex were conducted for 100 ns, followed by root-mean-square deviation (RMSD) analysis. (**C**) The MD simulation (RMSF analysis) of the AlaRS mutant/Tanshinone I complex for 100 ns.

A deeper understanding of the interaction mechanisms between the small molecule drug Tanshinone I and several AlaRS mutants associated with CMT disease was achieved through molecular docking analyses of five aminoacylation domain mutants with Tanshinone I. The docking experiments revealed that Tanshinone I shares a conserved binding mode across these mutants, suggesting that it may influence their function through specific binding sites and similar mechanisms of action (Figure 6A). Tanshinone I interacts with four residues in the AlaRS mutants: Arg77, Phe98, Asn216, and Arg246. The interactions between Arg77 and Arg246 are mediated by hydrogen bonds, while a hydrophobic interaction occurs between Asn216 and Tanshinone I. In particular, the phenylalanine residue at position 98 (Phe98) forms a π-π stacking interaction with Tanshinone I. This specific interaction is crucial for stabilizing the binding of Tanshinone I to the mutant protein, as π-π stacking typically occurs between aromatic rings and contributes to the overall binding affinity.

In order to evaluate the stability of Tanshinone I at the binding sites of the mutant proteins and to validate the reliability of the docking results, we performed molecular dynamics (MD) simulations. These simulations allow for the analysis of the conformational fluctuations, structural dynamics, and stability of protein–ligand complexes. By calculating the root-mean-square deviation (RMSD) values of the protein–ligand complexes after docking, we assessed whether significant structural changes occurred during the simulation. The RMSD analysis showed that the fluctuations of the mutant in the complex were greater than when it existed in isolation, due to conformational changes within the complex (Figure 6B). Additionally, we performed root-mean-square fluctuation (RMSF) analysis to identify flexible regions within the protein–ligand complex. Higher RMSF values typically indicate greater flexibility and lower structural stability. Our calculations for the AlaRS^R329H^/Tanshinone I complex revealed that certain residues had RMSF values exceeding 0.6 nm; however, the overall structure remained stable (Figure 6C).

Collectively, molecular docking analyses and molecular dynamics simulations revealed that Tanshinone I interacts with key residues in several AlaRS mutants, demonstrating a conserved binding mode that may influence their function. The stability of the AlaRS^R329H^/Tanshinone I complex was further confirmed through RMSD and RMSF analyses, which indicated greater fluctuations in the complex compared to the isolated mutant, although the overall structure remained stable.

### 2.6. Tanshinone I Disrupts the Binding of AlaRS Mutants with Nrp1

**Figure 7:**
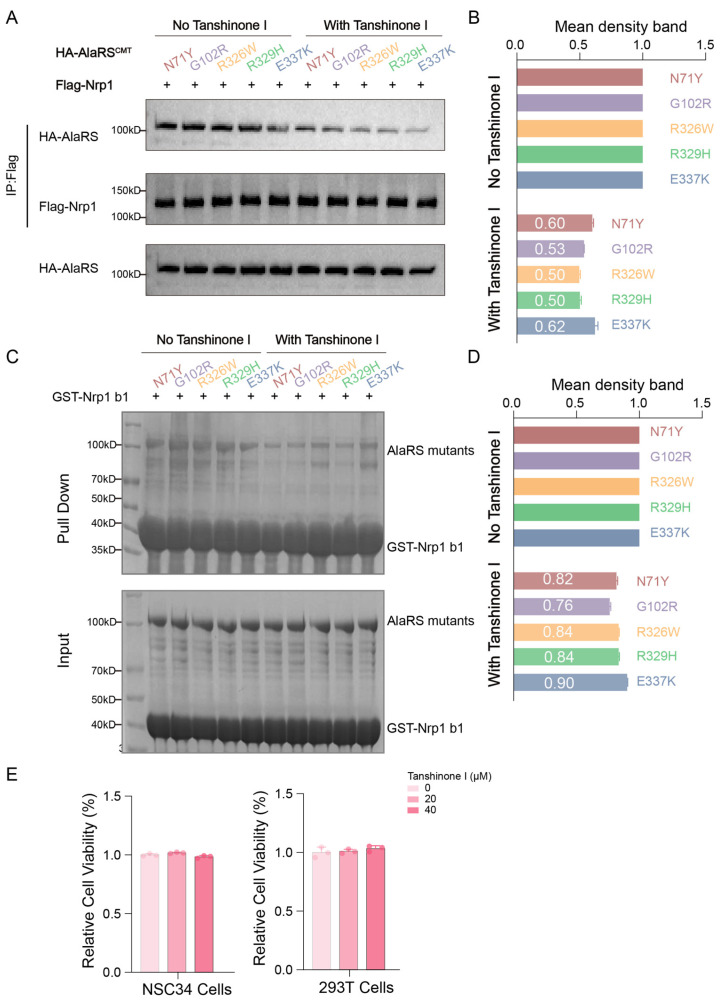
The impact of Tanshinone I on the interaction between AlaRS mutants and Nrp1. (**A**) Co-IP assays were conducted to assess the binding between HA-AlaRS mutants and Nrp1, both with and without treatment with 20 µM Tanshinone I for 24 h. (**B**) Relative quantification of the AlaRS mutants immunoprecipitated from the Co-IP experiments post-Tanshinone I treatment was performed using ImageJ, with the averages from three experimental repeats displayed in the figure. (**C**) Pull down assay was performed to detect the binding between AlaRS mutants and GST-Nrp1 b1 with or without 20 µM Tanshinone I. (**D**) Relative quantification statistics of the AlaRS mutants pulled down from the GST pull down experiments after Tanshinone I treatment were performed using ImageJ. The averages of the three experimental repeats are presented in the figure. (**E**) The cell viability of 293T and NSC34 cells treated with different concentrations of Tanshinone I was assessed using the CCK-8 assay over a 24 h period.

To further investigate whether Tanshinone I-induced conformational changes in AlaRS CMT mutants affect their interaction with Nrp1, Co-IP experiments were performed in cells treated with Tanshinone I. By comparing Co-IP results obtained before and after treatment, we assessed the influence of the drug on the binding strength and mode between the AlaRS CMT mutants and Nrp1. In transfected cells, treatment with 20 μM Tanshinone I for 24 h resulted in a significant reduction in the number of CMT mutants bound to Nrp1 (Figure 7A,B), indicating that Tanshinone I effectively affects this binding. To confirm the direct effect of Tanshinone I on the interaction between AlaRS CMT mutants and Nrp1, pull-down experiments were performed. These experiments showed that the addition of Tanshinone I significantly inhibited the direct binding between AlaRS mutants and Nrp1-b1 (Figure 7C,D). This finding suggests that Tanshinone I effectively disrupts this pathological interaction, possibly by inducing conformational changes in the AlaRS mutants that prevent their abnormal association with Nrp1. Given that the interaction between AlaRS mutants and Nrp1 is associated with disease progression in CMT, the ability of Tanshinone I to disrupt this binding highlights its potential to intervene in the pathogenic mechanisms of these diseases.

Further validation of the safety and efficacy of Tanshinone I involved conducting cytotoxicity assays to ensure minimal toxicity to normal cells, while effectively inhibiting the abnormal interaction between AlaRS CMT mutants and Nrp1. We used the CCK-8 assay to evaluate the effects of Tanshinone I on 293T and NSC34 cells. Cells were treated with different concentrations of Tanshinone I for 24 h, followed by incubation with CCK-8 reagent. The results showed that Tanshinone I, even at a concentration of 40 μM, did not significantly affect the viability of either cell type (Figure 7E). This finding suggests that Tanshinone I effectively disrupts the abnormal binding between AlaRS mutants and Nrp1, while having minimal effects on normal cells, supporting its potential safety as a therapeutic agent. Collectively, these results reinforce the potential of Tanshinone I to address the pathological mechanisms associated with CMT-related mutants and highlight its promising prospects for clinical application.

**Figure 8:**
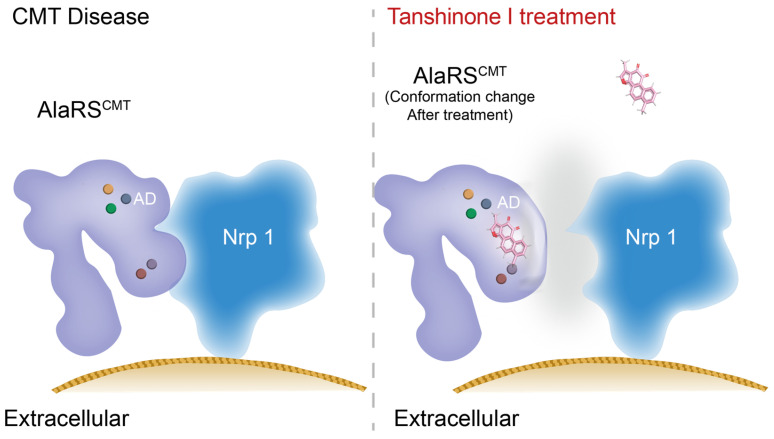
Tanshinone I as a therapeutic candidate for CMT2N via the targeting of AlaRS CMT mutant proteins. The abnormal binding between AlaRS CMT mutants and Nrp1 inhibits the interaction between Nrp1 and its receptor, thereby suppressing the subsequent signaling response. Our screened compound, Tanshinone I, binds to AlaRS CMT mutants, alters their conformation, and inhibits the abnormal binding to Nrp1, thereby allowing Nrp1 to perform its function.

In summary, Tanshinone I was identified as a compound that selectively binds to AlaRS mutations associated with CMT. Our findings demonstrate that Tanshinone I effectively disrupts the interaction between AlaRS mutants and Nrp1, thereby allowing Nrp1 to fulfill its functional role (Figure 8).

## 3. Materials and Methods

### 3.1. Cell Lines and Cell Culture

NSC34 cells were purchased from Guangzhou Jennio Biotech Co., Ltd. (Guangzhou, China) and 293T cells were preserved in our laboratory. NSC34 and 293T cells were cultured in Dulbecco’s Modified Eagle Medium (DMEM, Gibco, Carlsbad, CA, USA) supplemented with 1% penicillin/streptomycin and 10% Fetal Bovine Serum (FBS, Gibco, Carlsbad, CA, USA). All cell lines used in this study were incubated at 37 °C in a humidified incubator containing 5% CO_2_.

### 3.2. Western Blot Assay

Total protein was extracted from cells using RIPA buffer (50 mM Tris-HCl pH 7.4, 150 mM NaCl, 1% NP40, 1 mM EDTA) supplemented with a protease and phosphatase inhibitor cocktail (Roche, Basel, Switzerland). After SDS-PAGE, the protein on the gel was transferred to a PVDF membrane (Bio-Rad, Hercules, CA, USA) and then blocked with 5% non-fat dry milk in TBST for 1 h. Then, the membranes were incubated with the indicated primary antibodies and corresponding HRP-conjugated secondary antibodies. Signals were detected using the Bio-Rad ChemiDoc system (Bio-Rad, Shanghai, China). The following antibodies were used: Flag (Ray antibody biotech, Beijing, China, 1:3000), HA (Ray antibody biotech, Beijing, China, 1:3000), and GAPDH (Ray antibody biotech, Beijing, China, 1:5000).

### 3.3. Co-Immunoprecipitation (Co-IP)

To detect the binding of Nrp1 and AlaRS mutants in cells, whole-cell lysates (500 μg) were treated overnight with 2 μg of anti-Flag antibody in RIPA buffer. Next, Protein A/G agarose beads (#sc-2003, Santa Cruz Biotechnology, Santa Cruz, CA, USA) were applied to the lysates and the samples were further treated for 8–12 h with rotation. Samples were washed three times with RIPA buffer. Subsequently, the immunoprecipitations were resuspended into 2 × SDS-loading buffer. The samples were then boiled for 10 min and detected using Western blot. The experiment was repeated three times.

### 3.4. Expression and Purification of CMT-Related AlaRS Mutants

To further conduct in vitro experiments on the mutant proteins, AlaRS mutants were cloned into the pET-21a vector and transformed into BL21 (DE3) cells. The transformed cells were initially cultured overnight in LB medium containing 100 µg/mL ampicillin until saturation. The saturated culture was then diluted 1:200 in fresh LB medium and incubated at 37 °C. When the optical density (OD600) reached 0.5, protein expression was induced by adding isopropyl β-D-thiogalactopyranoside (IPTG) to a final concentration of 0.3 mM. Following induction, the cells were incubated at 16 °C for 20 h. Cells were then harvested by centrifugation at 4 °C, 4000 rpm for 45 min. The resulting cell pellet was resuspended in lysis buffer (20 mM Tris-HCl, 300 mM NaCl, 5 mM imidazole, 1 mM phenylmethylsulfonyl fluoride (PMSF), pH 8.0). Cell lysis was performed using an ultrasonic cell disruptor, and the lysate was clarified by centrifugation at 4 °C, 20,000 rpm, for 1 h. The proteins were then purified through a series of steps: first, using Ni-NTA beads (Cytiva, Marlborough, MA, USA), followed by ion-exchange chromatography on a HiTrap Q HP column (Cytiva, Marlborough, MA, USA), and, finally, size-exclusion chromatography on a HiLoad 16/60 Superdex 200 prep grade column (Cytiva, Marlborough, MA, USA). All purification steps were conducted at 4 °C or on ice to preserve protein stability and activity.

### 3.5. Thermal Shift Assay

To assess the impact of small molecules on the thermal stability of mutant proteins, thermal shift assays were performed using a StepOnePlus 7 Flex Real-Time Cycler (Applied Biosystems, Waltham, MA, USA) with the Protein Thermal Shift™ Kit dye (Thermo Fisher Scientific, Waltham, MA, USA). Each well of a 96-well Optical Reaction Plate (Applied Biosystems) contained 14 μL of thermal shift buffer, 2 μL of diluted thermal shift dye (8×), 2 μL of a 1 mM small molecule solution, and 2 μL of 1 mg/mL protein. Plates were incubated at 4 °C for 1 h before being heated from 25 °C to 95 °C at 1 °C/min. The fluorescence signal of SYPRO orange (excitation/emission: 490/530 nm) was recorded at 30 s intervals to monitor protein denaturation. The melting temperature (Tm), defined as the midpoint of the unfolding transition, was determined from the fluorescence–temperature curve. ΔTm was calculated as the difference in Tm between AlaRS^R329H^ treated with compounds and the control. Experiments were conducted in triplicate for accuracy. The experiment was repeated three times.

### 3.6. Bio-Layer Interferometry Assay

To further quantify the binding of small molecules to mutant proteins, binding experiments with AlaRS^R329H^ proteins and small molecules were conducted using an Octet R8 system (ForteBio, Fremont, CA, USA). Purified His-tagged AlaRS^R329H^ proteins (500 μg/mL) were captured on Ni-NTA biosensors (ForteBio, Fremont, CA, USA), achieving a saturation response of 4–5 nm after 300 s. The biosensors were then transferred to Octet buffer (PBS with 0.01% Tween 20) for 180 s to remove loosely bound, nonspecific proteins and to establish a stable baseline. Following this, the sensors were immersed in diluted Tanshinone I (2.5–100 μM in assay buffer) for 120 s to record the association signal, and then transferred back into Octet buffer to monitor the dissociation signal for 120 s. Reference wells containing buffer instead of Tanshinone I were included to correct for baseline shifts. Additionally, a parallel set of Ni-NTA sensors in buffer served as negative reference controls to account for nonspecific binding of the compounds to the biosensor surface. The data were processed using a double reference subtraction protocol to eliminate nonspecific signals, background noise, and signal drifts caused by biosensor variability. All assays were performed in 96-well black plates with a total volume of 200 μL per well at 30 °C. Data analysis, including correction and curve fitting, was carried out using Octet^®^ Analysis Studio 12.2.2.26. The experiment was repeated three times.

### 3.7. Enzyme Inhibition Assay

The ATP consumption assay was used to evaluate the enzymatic inhibitory activity of the compounds. A 30 μL reaction mixture, containing 1 μM AlaRS mutants, 25 μM ATP, 50 μM L-alanine, 30 mM HEPES (pH 7.5), 150 mM NaCl, 30 mM KCl, 40 mM MgCl_2_, 0.1% BSA, and varying concentrations of Tanshinone I, was incubated at room temperature for 15 min. After incubation, 10 μL of the mixture was transferred to a 384-well microplate, and AlaRS mutant activity was measured by adding 10 μL of the ATP Assay Kit (#S0026, Beyotime, Shanghai, China). Luminescence was recorded using a Synergy HTX multimode microplate reader (BioTek Inc., Winooski, VT, USA). The inhibitory rate was calculated from three independent assays to ensure the accurate assessment of Tanshinone I’s inhibitory activity. The experiment was repeated three times.

### 3.8. Molecular Docking

To understand binding affinities and identify potential binding sites, we conducted molecular docking. Autodock Vina v.1.2.2 software was used for molecular docking [24]. The structure of the AlaRS^R329H^ receptor macromolecule and ligand Tanshinone I were prepared before docking. The macromolecule receptor was modified by removing water molecules and adding polar hydrogen atoms, and then, the semiflexible docking method was used to generate the model. Molecular visualization and analysis were performed using PyMol (https://pymol.org, accessed on 20 August 2024).

### 3.9. Molecular Dynamics Simulation

In this experiment, a protein–small molecule complex composed of AlaRS^R329H^ and Tanshinone I was simulated using the Gromacs program. The process began with molecular preparation, where we generated topology files and simulation boxes using the pdb2gmx and gmx editconf commands after importing the structure files of the protein and small molecule. Next, energy minimization was performed using the gmx grompp and gmx mdrun commands to optimize the structure of the complex. Molecular dynamics (MD) simulation was then carried out for 100 ns under conditions of 300 K and 1 bar, using the Amber99sb-ildn force field and Tip3p water model.

### 3.10. Glutathione S-Transferase (GST) Pull Down Assay

To detect the direct interactions between the mutant proteins and Nrp1 in vitro, we performed a Glutathione S-transferase (GST) pull-down assay. AlaRS mutants fused with 6 × His were purified with Ni-NTA beads, followed by chromatography on a HiTrap Q HP column. Nrp1 b1 fragments fused with GST were purified with Glutathione Beads (#SA008100, Smart-Lifesciences, Changzhou, China). For GST pull-down assays, 1000 μg of each GST-fused Nrp1 b1 fragment was mixed with 200 μg of 6 × His-tagged fusion protein in binding buffer (50 mM Tris-HCl pH 7.5, 20 mM NaCl, 5% glycerol, 5 mM DTT with 0.1% BSA) at 4 °C overnight. The mixture was next incubated with 30 μL glutathione beads at 4 °C with constant rotation for 2–4 h. Subsequently, the bead/protein complex was washed three times, and eluted with 30 μL 2 × SDS sample buffer. The samples were then subjected to SDS-PAGE analysis. The experiment was repeated three times.

### 3.11. Cell Viability Measurement (CCK-8 Assay)

The Cell Counting Kit-8 (CCK-8) assay was performed to assess cell viability following the manufacturer’s instructions. Briefly, cells were seeded at an initial density of 5 × 10^3^ cells per well in a 96-well plate and incubated overnight. The following day, the cells were treated with various concentrations of Tanshinone I. After 24 h of treatment, 10 μL of CCK-8 reagent (GlpBio, Montclair, CA, USA) was added to each well, and the plate was incubated at 37 °C with 5% CO_2_ for 1.5 h. Absorbance was then measured at 450 nm using a multimode microplate reader. The experiment was repeated three times.

### 3.12. Statistical Analysis

Statistical analyses for all data in this study were conducted using SPSS 25 or GraphPad Prism 8.0 software. Descriptive statistics for numerical variables are presented as mean ± standard deviation (SD) or standard error of the mean (SEM). Differences between the means of two groups were compared using *t*-tests, with a significance level set at *p* < 0.05.

## 4. Discussion

Accumulating evidence from patient samples, genetic studies in animal models, and protein conformational analyses suggests that specific aminoacyl-tRNA synthetase (aaRS) variants in CMT disease not only participate in aminoacylation, but also confer a toxic gain-of-function [6]. In this context, high-throughput screening techniques can identify small molecule drugs that effectively disrupt the abnormal interactions between these variants and their targets, such as neuronal receptors.

In this study, we investigated the pathological impacts of AlaRS mutations associated with CMT2N, and evaluated the potential of small molecule drugs in mitigating these effects. Our findings highlighted the structural and functional consequences of AlaRS mutations related to CMT2N, assessing the therapeutic potential of small molecules like Tanshinone I. By developing small molecules that alter the conformation of these mutant proteins and inhibit their aberrant behavior, we can fundamentally disrupt or reverse disease progression. Targeting the toxic functions induced by mutations allows for a precise treatment approach, focusing on correcting specific pathogenic mechanisms rather than broadly enhancing cellular function. While current medications for CMT alleviate symptoms, protein-based therapies targeting the toxic functions of mutations, due to their specificity and curative potential, are anticipated to be key directions for future CMT treatments.

Tanshinone I, a key active component in *Salvia miltiorrhiza* (Danshen), has gained considerable attention for its proven bioactivities, including antioxidative stress, the regulation of autophagy and apoptosis, and inflammation inhibition [25]. It has demonstrated promising therapeutic potential in Parkinson’s disease (PD) research, effectively enhancing dopaminergic neuron health by reducing apoptosis and oxidative stress while increasing nuclear factor erythroid 2-related factor 2 (Nrf2) and its associated antioxidant gene expression [26]. In the context of neuronal damage caused by CMT-related mutations, Tanshinone I may alleviate oxidative stress, protect neurons, and enhance cellular health through similar mechanisms. Additionally, many CMT mutations lead to protein dysfunction or toxic gain-of-function; thus, Tanshinone I’s neuroprotective effects may extend beyond symptom relief by blocking the interaction between the CMT AlaRS mutant and Nrp1, fundamentally improving CMT pathology. Given its successful application in PD, Tanshinone I holds promise as an effective therapeutic agent for reducing nerve damage and ameliorating symptoms in CMT disease.

Despite the valuable insights gained from our study, several limitations warrant further discussion. Firstly, our research predominantly relied on in vitro experiments and cellular analyses to assess mutant functionality and small molecule effects. While these experiments provide crucial information, the absence of validation in animal models represents a significant limitation. Additionally, our focus on a limited number of specific AlaRS mutants and their interactions with Nrp1 may overlook other mutants or combinations with different pathological mechanisms and therapeutic responses. Therefore, future research should include a comprehensive analysis of additional AlaRS mutants to identify other potential targets or therapeutic strategies. Moreover, subsequent studies should incorporate animal models, long-term toxicity assessments, and analyses of a broader range of mutants to validate our findings further and facilitate the translation of small molecule drugs into clinical applications.

## Figures and Tables

**Table 1:** The four small positive hits.

Compounds	Structure	ΔTm (℃) ^a^	K_D_ (μM) ^b^	Relative Activity (%) ^c^
Atazanavir sulfate	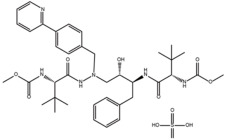	4.0	28.6 ± 9.9	96.8
Tanshinone I	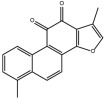	3.1	5.1 ± 1.9	26.2
Hederacolchiside A1	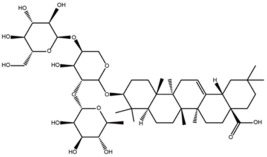	4.3	10.1 ± 0.5	69.6
Hexylresorcinol	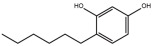	5.5	26.3 ± 9.9	79.8

^a^ ΔTm is the shift between the Tm values of AlaRS^R329H^ with and without compounds. ^b^ The K_D_ values measured by BLI are presented as the mean ± SD, with the experiment repeated three times. ^c^ Relative enzyme activity represents the ratio of the enzymatic activity of 1 μM AlaRS^R329H^ at 20 μM compounds. The data are the average of two independent experiments.

## Data Availability

Data are contained within the article and Supplementary Materials.

## Supplementary Materials

The following supporting information can be downloaded at: https://www.mdpi.com/article/10.3390/ijms252011184/s1.
